# A multicenter randomized controlled effectiveness trial of transdiagnostic treatment for adolescents with subthreshold anxiety and depressive symptoms

**DOI:** 10.1017/S0033291726105443

**Published:** 2026-09-04

**Authors:** Eduardo Fonseca-Pedrero, Alfonso Pérez-Esteban, Adriana Díez-Gómez, Alicia Pérez-Albéniz, Susana Al-Halabí, Beatriz Lucas-Molina, Martin Debbané

**Affiliations:** 1 Universidad of La Rioja, Spain; 2 Universidad of Oviedo, Spain; 3 Universitat of Valencia, Spain; 4 Universite of Geneve, Switzerland

**Keywords:** adolescence, anxiety, depression, indicated prevention, mental health, transdiagnostic psychological treatment

## Abstract

**Background:**

Depression and anxiety disorders and symptoms are among the leading causes of disability and global disease burden in young people. Scalable, unified interventions are required to address subthreshold emotional symptoms in adolescents as a preventive strategy. This study evaluated the effectiveness of a transdiagnostic treatment for adolescents with subthreshold emotional symptoms.

**Methods:**

A multicenter, two-arm, randomized controlled trial was conducted. The Unified Protocol for Adolescents (UP-A) was compared with an active control consisting of protocolized progressive relaxation. A total of 676 adolescents (72.6% female; *M*_age_ = 13.77; *SD* = 1.39; range = 12–18 years) participated in the study. Primary outcomes were self-reported depressive and anxiety symptoms at postintervention, 6 months, and 12 months. The trial is registered with ClinicalTrials.gov (NCT05322642).

**Results:**

Three hundred seventy-eight participants were allocated to the UP-A group and 298 to the control group. Adherence was high in both conditions (≈78% attended >70% of sessions). Both interventions showed significant reductions in depressive and anxiety symptoms over time, with no significant between-group differences. At 12 months, the UP-A group showed marginally lower depressive symptoms and neuroticism. Significant treatment effects favoring the UP-A were observed for hyperactivity, conduct problems, and total mental health difficulties. Improvements were maintained across most outcomes at both follow-ups.

**Conclusions:**

The comparable improvements observed across both conditions suggest that structured, group-based school interventions may help reduce emotional symptoms in adolescents with subthreshold presentations, rather than reflecting the superiority of a specific protocol. These findings support their potential for broad dissemination within stepped-care frameworks and for enhancing accessibility and real-world effectiveness of evidence-based treatments.

## Introduction

Adolescence is a pivotal developmental period characterized by rapid biological, psychological, and social transitions that increase vulnerability to emotional problems and the emergence of mental disorders. At the same time, it offers a unique window for learning, identity formation, and resilience-building that shapes long-term well-being. Across the life course, nearly a quarter (24.85%) of all Years Lived with Disability attributable to mental disorders arises before age 25 years (Kieling et al., 2024). The onset of mental disorders typically occurs during youth, with incidence peaking at a mean age of 14.5 years. Moreover, an estimated 34.6% of individuals experience the onset of a mental disorder before the age of 14 (Solmi et al., 2022). Emotional disorders, as well as their subthreshold presentations, are associated with considerable distress and substantial impairments in personal, academic, family, health, and social functioning (Arrondo et al., 2022; GBD 2019 Mental Disorders Collaborators, 2022; McGinty et al., 2024; McGorry et al., 2024).

Inadequate attention to adolescent mental health may result in substantial adverse consequences across both the short and long term, ultimately diminishing young people’s prospects for attaining healthy, productive, and fulfilling adult lives. Consequently, scalable and unified interventions are needed to address subthreshold emotional symptoms in adolescents as a preventive strategy beyond traditional clinical settings. Within this framework, clinical staging models conceptualize mental disorders as dynamic processes that evolve from subthreshold manifestations to fully developed syndromes, underscoring the importance of early, proportionate, and adaptive intervention to optimize long-term outcomes (McGorry & Mei, 2021).

Evidence-based preventive interventions constitute an effective strategy for supporting adolescents at risk of developing emotional problems before full clinical manifestations emerge. Although many empirically supported treatments (ESTs) have demonstrated efficacy in reducing internalizing symptoms among youth (Sanchez et al., 2018; Weisz & Kazdin, 2017), social, health, and educational systems remain substantially under-resourced, resulting in persistent global treatment gaps. The transdiagnostic approach to psychological disorders puts forward a novel and useful approach that could prevent the development of later mental health problems, enhance treatment responsiveness and recovery rates, and ultimately reduce youth suffering (Dalgleish et al., 2020; Farchione et al., 2024; Newby et al., 2015). Transdiagnostic interventions typically target shared risk factors or commonalities underlying multiple mental disorders, efficiently addressing the needs of those who present with more complex patterns of comorbidity (Barlow et al., 2011). Critically, transdiagnostic approaches can facilitate the dissemination of ESTs, orienting toward implementations across settings and populations.

Transdiagnostic interventions have proven effective in addressing depressive and anxiety disorders in both adults and adolescents (Carlucci et al., 2021; Cassiello-Robbins et al., 2020; Cuijpers et al., 2023; Farchione et al., 2024; Newby et al., 2015; Sakiris & Berle, 2019; Schaeuffele et al., 2024). The Unified Protocol for Transdiagnostic Treatment of Emotional Disorders in Adults (UP) represents a gold standard of transdiagnostic interventions (Barlow et al., 2011). The UP has been adapted for youth as the UP-C (children) and Unified Protocol for Adolescents (UP-A) (adolescents), providing a transdiagnostic, emotion-focused cognitive behavioral intervention that addresses temperamental vulnerabilities such as neuroticism and emotion dysregulation.

The UP-A is designed for adolescents aged 12–17 years with any primary emotional disorder, including anxiety, depression, obsessive–compulsive, and stress-related conditions. Techniques draw on core Cognitive Behavioral Therapy strategies while integrating motivational enhancement, awareness building, and mindfulness (Ehrenreich-May et al., 2018). Evidence from randomized clinical trials supports its efficacy across cultures and delivery formats, and studies consistently show reductions in emotional distress and improvements in adaptive psychological processes in clinical, subthreshold, and non-clinical populations (Ehrenreich-May et al., 2025; Espinosa et al., 2024; García-Escalera et al., 2020; Vivas-Fernandez et al., 2025). Unified Protocols have been developed to overcome barriers to the dissemination of evidence-based psychological treatments. However, few preliminary studies have examined the UP-A in a large sample of adolescents at high risk for emotional disorders in non-clinical settings. In addition, they often fail to incorporate measures of psychological strengths – critical for supporting and maintaining mental health – as well as parent-reported assessments. Furthermore, they seldom examine the durability of effects, or whether treatment gains are maintained at long-term follow-up (McGinty et al., 2024).

In this context, the present study evaluated the effectiveness and duration of the potential benefits of the UP-A for adolescents with subthreshold emotional symptoms in non-clinical settings. We hypothesized that the UP-A would be more effective than the active control group in reducing emotional symptoms and transdiagnostic dimensions of emotional disorders at the acute outcome, as well as at 6- and 12-month follow-ups. In addition, we hypothesized that adolescents randomly assigned to the UP-A condition would exhibit greater improvements than those receiving the active control condition in psychological strengths, quality of life, and suicidal behavior.

## Methods

### Study protocol

The Evidence-based Psychology in Educational Contexts (PSICE) study was a randomized controlled trial (RCT) registered (ClinicalTrials.gov, identifier: NCT05322642). The design encompasses a larger two-armed RCT comparing active control (Progressive Relaxation Training) and experimental (UP-A). Psychologists and adolescents were randomized, and treatment outcome measures were collected by multiple informants at posttreatment and at 6-month and 12-month follow-ups. The PSICE study was approved by the ethics committees of the Government of La Rioja and the University of La Rioja (CEImLAR, PI552).

### Participants

Participants were recruited from nine provinces distributed across different regions of Spain, providing a broad geographical coverage. The screening phase included a total of 8,746 students from 85 schools and 532 classrooms.

The students attended various public and charter educational centers for compulsory secondary education and vocational training, representing diverse socioeconomic backgrounds. Sex assigned at birth was collected through self-report questionnaires, with response options male, female, and intersex. Ethnic or racial characteristics were not assessed; only nationality was recorded.

Inclusion criteria for the adolescents were (a) being 12–18 years old; (b) presenting moderate symptom severity in both the PHQ-9 and GAD-7 (between 10 and 15 points); and (c) having adequate proficiency in Spanish. The exclusion criteria were: (a) having been diagnosed with a mental disorder or alcohol and/or substance dependence disorder and/or high risk of suicidal behaviors; (b) presenting any medical illness or condition that could interfere with participation; (c) receiving ongoing psychological or psychiatric treatment; or (d) lacking informed consent.

All the assessments were administered collectively through personal computers, in groups of 8–12 students, during regular school hours in a classroom specially prepared for this purpose. No incentive was provided for their participation. Participants were informed about the confidentiality of their responses and of the voluntary nature of their participation. Informed consent was obtained from all participants. For more detailed information on the PSICE protocol, see Fonseca-Pedrero, Pérez-Albéniz et al. (2023).

### Treatment

#### Experimental treatment

UP-A. An adapted version of the UP-A was used to ensure its feasibility in educational contexts and its suitability for adolescents with emotional symptoms, rather than clinically diagnosed emotional disorders (García-Escalera et al., 2020). The Spanish version of the UP-A is largely equivalent to the original and includes only minor cultural adaptations in the worksheets. The PSICE protocol consisted of ten 50-minute sessions of the UP-A, delivered in small groups (8–12 students) within school settings (see Supplementary Table S1). All participants received a copy of the Spanish UP-A patient manual. The module ‘Parenting the Emotional Adolescent’ was also included as part of this preventive adaptation.

#### Active control group

Progressive relaxation. This group received ten sessions based on the classic procedure developed by Bernstein and Borkovec (1973) (see Supplementary Table S2). Each 50-minute session focused on progressive muscle relaxation as a stress-reduction strategy and was delivered in small groups (8–12 students) within school settings. The program was standardized using a project-specific manual.

The professionals who implemented the interventions had specific prior training in the UP-A protocol, demonstrated experience working with adolescents, and received ongoing supervision from the technical team at the University of La Rioja. This training and support system ensured both fidelity to the protocol and high-quality in the delivery of the intervention.

### Randomization and masking

A computer-generated allocation sequence was used to randomly assign participants to either the experimental group or the control group in a 1:1 ratio within each educational center. The randomization process was carried out by an independent researcher who was not involved in the assessment or the intervention. Allocation was communicated to participants by the school psychologist. The participants were blinded to the treatment allocation.

### Measurement instruments

*The primary outcomes* were symptoms of depression and anxiety. The Patient Health Questionnaire-9 (PHQ-9) is a self-report used to explore the presence of major depressive disorder symptoms in the past 2 weeks. The answers to each PHQ-9 item are rated on a 4-point Likert response scale (0 = *not at all*; 3 = *nearly every day*). Cut-off points are: 0–5 = minimal, 6–9 = mild, 10–14 = moderate, 15–19 = moderately severe, and 20–27 = severe. The validated Spanish version of PHQ-9 was used in the present study (Fonseca-Pedrero et al., 2023). The Generalized Anxiety Disorder 7-item Scale (GAD-7) assesses the presence of generalized anxiety disorder symptoms in the past 2 weeks. The answers are rated on a 4-point Likert response scale (0 = *not at all*; 3 = *nearly every day*). Total scores ranging from 0 to 21 may be classified into four severity groups: minimal (0–4), mild (5–9), moderate (10–14), and severe (15–21). The Spanish version of the GAD-7 scores has shown adequate psychometric properties among adolescents (Casares et al., 2024).

*The secondary outcomes* were transdiagnostic dimensions of emotional disorders, positive affect and negative affect, emotional and behavioral problems, suicidal behavior, health-related quality of life, and self-esteem.

The Multidimensional Emotional Disorder Inventory (MEDI) (Rosellini & Brown, 2019) is a self-report used to assess the transdiagnostic dimensions of emotional disorders. It consists of 49 items that evaluate nine dimensions of emotional disorders. The Positive and Negative Affect Schedule for Children-Short form (PANAS-C) (Ebesutani et al., 2012) is composed of two factors designed to measure Positive affect and Negative affect. It is made up of 10 items rated on a 5-point Likert scale and assesses how individuals have felt over the past few weeks. The Strengths and Difficulties Questionnaire (SDQ), in both self-report and parent-report forms (Goodman, 1997) it is a widely used instrument for the assessment of different emotional and behavioral problems. The SDQ comprises 25 statements distributed across five subscales: Emotional symptoms, Conduct problems, Hyperactivity, Peer relationships problems, and Prosocial behavior. The SENTIA-Brief scale (Díez-Gómez et al., 2021) is a screening designed to detect suicidal behavior in educational settings. It consists of five items with a dichotomous response format (0 = no; 1 = yes), where higher scores indicate greater severity. The KIDSCREEN-10 Index (KS-10 Index) (Ravens-Sieberer et al., 2010) is an instrument developed and validated to assess health-related quality of life in children and adolescents. It consists of ten questions on a 5-point Likert scale. The Rosenberg Self-Esteem Scale (RSS) (Rosenberg, 1965) allows for the assessment of participants’ self-esteem. It is a unidimensional scale composed of ten items, with a 4-point Likert response format. Academic performance: Academic performance was assessed using an ad hoc item, in which students reported their average grade from the previous trimester.

The Feasibility and Acceptability Questionnaire (FAQ) (García-Escalera et al., 2020) is a 12-item measure designed to assess participants’ satisfaction with the intervention which includes: program enjoyment, perceived learning, usefulness for coping with everyday challenges and emotions, willingness to recommend the program to others, and perceived gains in emotional knowledge. Items are rated using a combination of response formats, including dichotomous (Yes/No) options, 4-point Likert-type scales, and 10-point rating scales.

### Statistical analyses

First, sample size for the treatment phase was estimated with G*Power program. Assuming *α* = 0.05, Cohen’s *d* = 0.8, and 95% power, 42 participants per group (84 total) were required. Allowing 20% attrition, the final target sample was 105 participants (≈53 per group).

Second, chi-square tests were performed for categorical data and *F* tests (ANOVA) for continuous data to confirm that there were no significant differences between the groups at the start of the study in any of the sociodemographic or clinical variables. To examine potential attrition bias, additional analyses were conducted to examine whether baseline sociodemographic or primary clinical outcomes (depressive and anxiety symptoms) were associated with study completion.

Third, to evaluate the effect of the two treatment procedures and changes in dependent variables over time, generalized estimating equation (GEE) models were used (Wang, 2014). GEE models have been established as a powerful alternative for longitudinal analysis and have been widely used in randomized controlled trials due to their advantages over other established statistical procedures, including non-linear mixed effects models. GEE models can accommodate missing data under the assumption that they are missing completely at random or missing at random. Given that GEE models are robust to missing values and considering that dropout was high in both groups (over 30%), no imputation method was applied in the primary analyses. This strategy follows the recommendations for the treatment of missing values in the analysis of longitudinal data. Although the autoregressive correlation structure, which establishes a first-order dependence between consecutive scores, is expected in longitudinal data we opted, to use an interchangeable working correlation structure in the analysis of all GEE models. In addition, previous research evaluating the effectiveness of the UP-A protocol has also used this working correlation matrix (Espinosa et al., 2024). The method of estimation of the scale parameters was maximum likelihood. The variables of sex and age at pretreatment were controlled in all GEE analyses.

To complement this approach and examine the robustness of the findings, sensitivity analyses were conducted for the primary outcomes (PHQ-9 and GAD-7) using multiple imputation. Forty imputed datasets were generated, including treatment group, time, age, sex, and all outcome variables across assessment points in the imputation model, and GEE analyses were then replicated across imputed datasets.

Pairwise comparisons with Bonferroni corrections were performed, and Cohen’s effect sizes (*d*) were calculated to quantify the effects of the interventions on each dependent variable. Effect sizes were interpreted following conventional cut-offs for Cohen’s *d* (≈0.20 = small, ≈0.50 = moderate, ≈0.80 = large). In this study, effects generally fell within the small-to-moderate range, which is considered clinically meaningful in preventive interventions with non-clinical adolescent samples.

In addition to determining clinically significant change, we identified treatment responders by calculating the percentage of participants who achieved a greater improvement of 30% or 50% on the PHQ-9 or GAD-7 scores between pretreatment and posttreatment, as well as between pretreatment and 12-month follow-up.

All analyses were performed using IBM SPSS Statistics software, version 26.0.

## Results

### Participant flow and baseline characteristics

**Figure 1.**
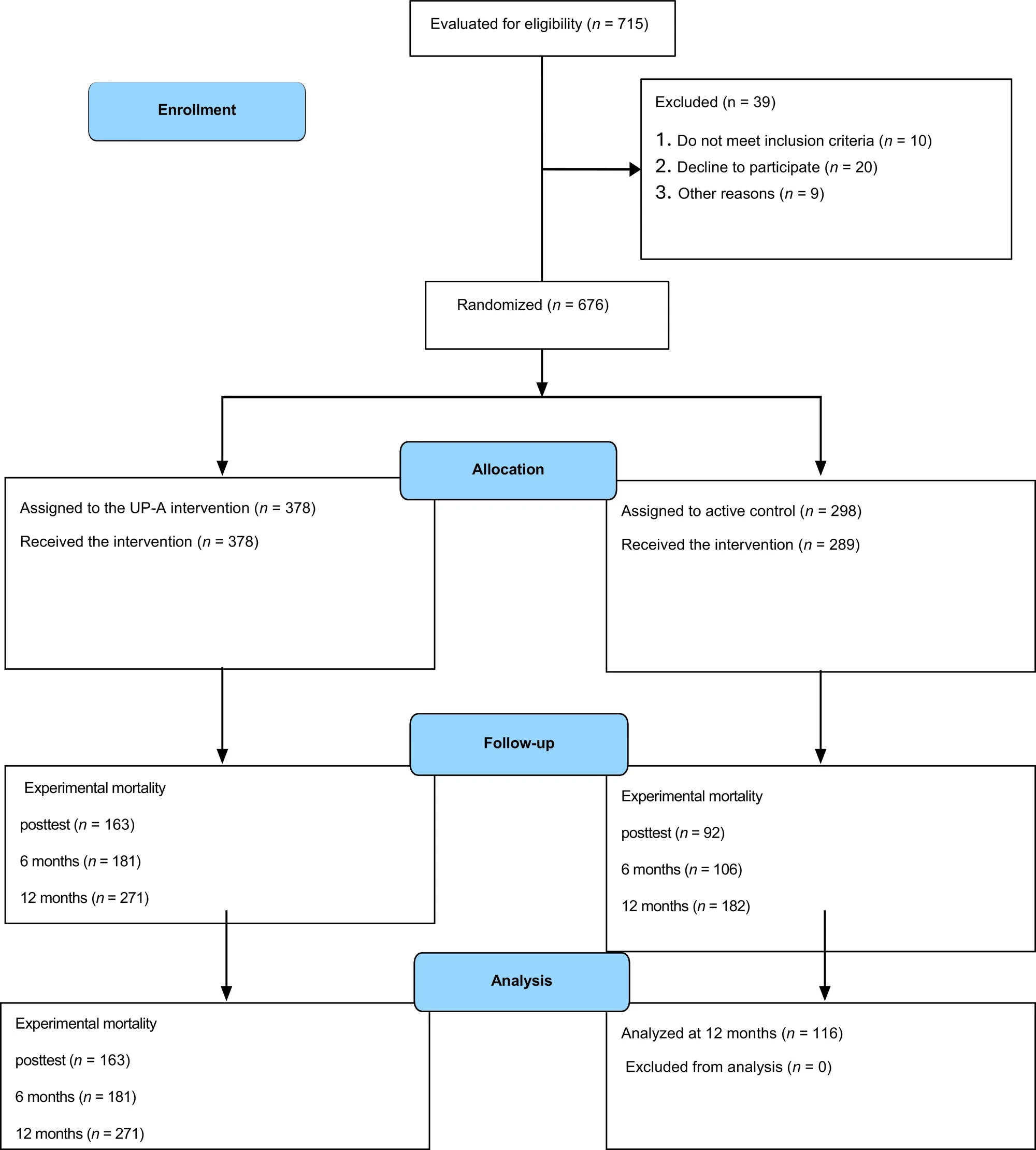
PSICE study CONSORT trial profile. Figure 1. long description.

Between September 1, 2022, and June 1, 2024, 8,746 students were screened, and 715 at-risk adolescents were eligible and consented to participate in the PSICE study. A total of 676 adolescents (72.6% female; *M*_age_ = 13.77; *SD* = 1.39; range = 11–18 years) participated in the study. Figure 1 depicts the PSICE trial profile and summarizes the participant flow according to CONSORT guidelines. Supplementary Table S3 presents the frequency and percentage of dropouts for both the UP-A and active control conditions at the different assessment points: before the intervention, immediately after the intervention, and at the 6- and 12-month follow-ups.

Regarding intervention adherence, 295 (78%) participants in the UP-A group and 233 (78.2%) in the active control group attended more than 70% of the intervention sessions. The average number of sessions attended in both groups was 8 (Median = 9; *SD* = 2).

No statistically significant differences were found between the groups at the start of the study in terms of sociodemographic and clinical characteristics (Table 1).

**Table 1.** Sociodemographic and clinical characteristics of the sample at baseline Table 1. long description.

	UP-A	Active control	Overall
	(*n* = 378)	(*n* = 298)	(*n* = 676)
Sociodemographic variables			
Age (*M* [*SD*])	13.78 (1.41)	13.75 (1.36)	13.77 (1.39)
Sex (*n* [% women])	277 (73%)	213 (71%)	490 (72%)
Nationality (*n* [% Spanish])	354 (94%)	268 (90%)	622 (92%)
Socioeconomic status (*M* [*SD*])	6.12 (1.90)	6.15 (1.95)	6.14 (1.92)
Clinical variables			
Mental health history (*n* [% ever had a mental health problem])	174 (46%)	127 (43%)	301 (45%)
Psychologist history (*n* [% ever consulted a psychologist])	130 (34%)	110 (37%)	240 (36%)

*Note*: M, mean; SD, standard deviation.

To examine potential attrition bias, baseline characteristics of completers and non-completers at the 12-month follow-up were compared (see Supplementary Table S5). Completers were younger at baseline in both conditions (*p* = 0.013), but no differences were observed in depressive or anxiety symptoms (PHQ-9, GAD-7) or other sociodemographic variables. This pattern suggests some age-related attrition, without evidence of bias in the primary clinical outcomes.

### Primary outcomes: Depression and anxiety symptoms

**Figure 2.**
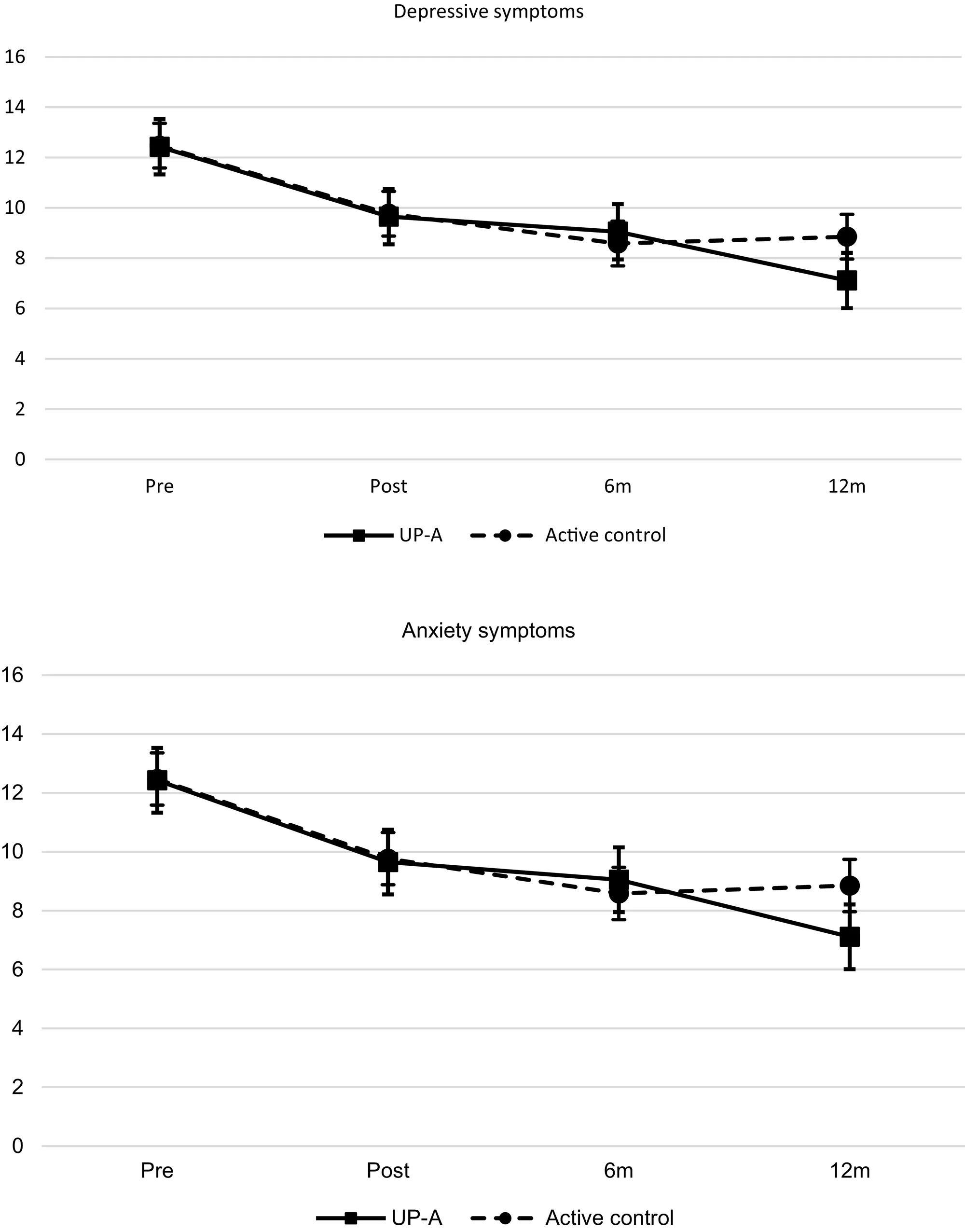
Mean scores on the depressive symptoms (PHQ-9) and anxiety symptoms (GAD-7) throughout the four assessment periods in the two intervention groups (UP-A versus active control). Figure 2. long description.

Regarding depressive symptoms, the GEE analysis revealed a statistically significant effect of Time (*p* < 0.0001), but no significant effect of Treatment Group (*p* = 0.41) or Time × Treatment Group interaction (*p* = 0.11) (see Table 2 and Figure 2). Pairwise comparisons indicated that both groups experienced a significant decrease in depressive symptoms from baseline to the end of treatment, as well as from baseline to the 6-month and 12-month follow-ups. However, while the UP-A group showed additional statistically significant differences between posttreatment and both follow-up assessments, the active control only maintained these differences between posttreatment and the 6-month follow-up. These within-group patterns should be interpreted cautiously, given the lack of a significant interaction effect. From posttreatment onwards, the UP-A group obtained lower scores on depressive symptoms compared to the active control group, with marginal significance at 12 months of follow-up (*p* = 0.056). Effect sizes were moderate to large for both groups.

**Table 2.** Descriptive statistics, GEE results for the effect of the intervention (UP-A versus active control) on PHQ-9 (depressive symptoms) and GAD-7 (anxiety symptoms), and effect sizes across the four time points Table 2. long description.

	Pretest		Postest		Posttest 6 m		Posttest 12 m		Wald’s Chi-square						*d* (Pre-Post)	*d* (Pre-6 m)	*d* (Pre-12 m)
	M	SD	M	SD	M	SD	M	SD	Time	*p* value	Group	*p* value	GxT	*p* value			
PHQ-9																	
UP-A	12.43	3.15	9.65	5.06	9.05	5.64	7.11	4.89	198.58	<0.0001	0.68	0.41	6.00	0.11	0.66	0.74	1.29
Control	12.47	3.43	9.77	5.36	8.58	5.83	8.85	6.48	–	–	–	–	–	–	0.60	0.81	0.70
GAD-7																	
UP-A	12.48	3.01	9.37	5.09	8.75	5.16	6.94	4.76	255.20	<0.0001	1.18	0.28	3.67	0.30	0.75	0.88	1.39
Control	12.57	2.98	9.49	4.64	8.57	5.08	8.41	5.83	–	–	–	–	–	–	0.79	0.96	0.90

*Note*: M, mean; SD, standard deviation; G, group; T, time.

Regarding anxiety symptoms, the GEE analysis showed a statistically significant effect of Time (*p* < 0.0001), but no significant effect of Treatment Group (*p* = 0.28), nor a significant interaction between Time × Treatment Group (*p* = 0.30) (see Table 2 and Figure 2). Pairwise comparisons revealed that the UP-A group showed a statistically significant decrease in anxiety symptoms from baseline to posttreatment, as well as from baseline to the 6- and 12-month follow-ups, with a significant reduction also found between the two follow-ups. For its part, the active control group also showed significant decreases between all-time points, except between the 6- and 12-month follow-ups. Overall, both groups showed a similar pattern of improvement over time.

Sensitivity analyses using multiple imputation yielded a pattern of results consistent with the primary analyses. Specifically, a significant effect of Time was observed across imputations (*p* < 0.001 in all models), whereas no consistent effects of treatment Group or Group × Time interaction emerged.

The significant change in PHQ-9 scores in the UP-A group was as follows: a 30% reduction from pretreatment to posttreatment was observed in 100 (46%) participants; a 50% reduction, in 51 (23.7%). A 30% reduction from pretreatment to 12-month follow-up was observed in 84 (78.5%) participants; and a 50% reduction in 48 (44.8%). A significant change in the GAD-7 score was observed in the UP-A group: a 30% reduction from pretreatment to posttreatment was found in 100 (46.5%) participants; a 50% reduction, in 66 (30.7%). A 30% reduction from pretreatment to the 12-month follow-up was observed in 74 (69.2%) participants, and a 50% reduction in 51 (47.7%).

### Secondary outcomes: Transdiagnostic emotional dimensions

The GEE analyses revealed a statistically significant effect of the Time factor in all MEDI transdiagnostic emotional dimensions (*p* < 0.0001). No significant Treatment Group effect or significant interaction between Time × Treatment Group was found in any of the nine MEDI dimensions. Effect sizes were small to moderate for both treatment groups (see Table 3 and Supplementary Figure S1). Pairwise comparisons revealed that MEDI dimensions improved significantly from pretreatment to posttreatment and at the 6- and 12-month follow-ups in both groups. For the Neuroticism dimension, pairwise comparisons between the UP-A group and the active control group revealed that the difference in means at the 12-month follow-up was marginally significant (*p* = 0.052).

**Table 3.** Descriptive statistics, GEE results for the effect of the intervention (UP-A versus active control) on the assessed dimensions of the MEDI and effect sizes across the four time points Table 3. long description.

	Pretest		Postest		Posttest 6 m		Posttest 12 m		Wald’s Chi-square						*d* (Pre-Post)	*d* (Pre-6 m)	*d* (Pre-12 m)
	M	SD	M	SD	M	SD	M	SD	Time	*p* value	Group	*p* value	GxT	*p* value			
Neuroticism																	
UP-A	21.48	8.00	19.22	8.79	17.97	9.53	15.66	8.92	85.25	<0.0001	1.56	0.21	2.53	0.52	0.26	0.39	0.67
Control	22.49	8.15	19.28	8.95	19.34	9.41	18.18	10.50	–	–	–	–	–	–	0.35	0.34	0.44
Positive temperament																	
UP-A	22.53	7.62	24.19	8.05	24.43	7.74	25.89	7.43	79.42	<0.0001	0.38	0.50	0.65	0.89	0.21	0.24	0.44
Control	21.58	7.61	23.47	8.27	24.32	7.95	25.09	8.83	–	–	–	–	–	–	0.22	0.33	0.40
Depressed mood																	
UP-A	15.39	8.73	12.20	8.78	10.54	9.01	9.13	7.74	105.76	<0.0001	0.99	0.32	1.64	0.65	0.36	0.54	0.74
Control	16.03	9.05	12.43	9.06	11.46	9.49	10.80	10.86	–	–	–	–	–	–	0.37	0.46	0.50
Autonomic arousal																	
UP-A	13.43	9.17	10.11	8.13	9.33	8.90	8.46	8.41	65.99	<0.0001	0.24	0.62	1.74	0.63	0.38	0.45	0.55
Control	13.07	9.04	10.60	9.14	9.22	9.42	9.32	9.98	–	–	–	–	–	–	0.26	0.39	0.37
Somatic anxiety																	
UP-A	14.63	7.11	14.44	7.81	13.49	6.68	12.65	6.78	13.34	0.004	0.77	0.38	1.08	0.78	0.02	0.16	0.28
Control	14.82	7.13	14.86	7.57	14.71	7.10	14.15	6.05	–	–	–	–	–	–	0.01	0.01	0.09
Social anxiety																	
UP-A	16.13	10.68	14.22	10.77	13.54	10.96	12.42	10.70	32.00	<0.0001	1.64	0.51	0.44	0.65	0.17	0.24	0.34
Control	16.52	10.47	15.70	11.22	14.09	10.72	14.45	11.06	–	–	–	–	–	–	0.07	0.22	0.18
Intrusive cognitions																	
UP-A	19.06	11.45	14.17	11.69	13.26	12.01	11.03	11.11	121.09	<0.0001	0.77	0.38	1.26	0.74	0.42	0.49	0.39
Control	19.67	11.64	15.66	11.94	13.75	12.01	13.22	12.75	–	–	–	–	–	–	0.32	0.47	0.50
Traumatic re-experiencing																	
UP-A	14.23	10.51	10.65	10.18	10.14	10.90	7.51	9.79	65.76	<0.0001	0.24	0.63	1.55	0.67	0.34	0.38	0.65
Control	13.98	10.30	11.19	10.76	9.59	10.48	8.58	9.93	–	–	–	–	–	–	0.25	0.40	0.50
Avoidance																	
UP-A	25.14	11.63	22.80	12.32	21.50	13.05	19.91	11.72	46.64	<0.0001	0.58	0.81	1.24	0.74	0.19	0.29	0.44
Control	24.50	11.86	23.76	12.49	21.67	12.16	20.72	11.81	–	–	–	–	–	–	0.06	0.22	0.30

*Note*: M, mean; SD, standard deviation; G, group; T, time.

### Secondary outcomes: Emotional and behavioral difficulties (self-report and parents’ version) and suicidal behavior

The GEE analyses revealed a significant effect of Time on scores in the five dimensions and in the Total difficulties score of the self-report version of the SDQ (see Table 4). In addition, a statistically significant effect of the Treatment Group was found for Hyperactivity (*p* = 0.025) and Total difficulties (*p* = 0.048), and a significant interaction between Time and Treatment Group for Peer relationships problems (Wald *χ* ^2^ = 8.92, *p* = 0.030). Cohen’s *d* effect sizes comparing pretreatment with posttreatment and follow-ups were small to large for the UP-A group and small to medium for the active control group. Pairwise comparisons showed significant improvements in Emotional Symptoms, Hyperactivity, and Total Difficulties scores from pretreatment to posttreatment and at the 6- and 12-month follow-ups in both groups, as well as in Peer relationship problems for the UP-A group. It is worth noting that in the UP-A condition, significant improvements continued to be observed in Hyperactivity (*p* = 0.045) and the Total Difficulties score (*p* = 0.002) between the 6- and 12-month follow-ups. Regarding the effect of the Treatment Group, at posttreatment, the UP-A group showed significantly lower scores on the Total Difficulties score (*p* = 0.047) compared to the active control group. Furthermore, at the 12-month follow-up, the UP-A group obtained significantly lower scores than the active control group on the Hyperactivity scale (*p* = 0.022).

**Table 4.** Descriptive statistics, GEE results for the effect of the intervention (UP-A versus active control) on the five dimensions and total SDQ difficulties score (self-report and parent versions) and effect sizes across the four time points Table 4. long description.

	Pretest		Postest		Posttest 6 m		Posttest 12 m		Wald’s Chi-square						*d* (Pre-Post)	*d* (Pre-6 m)	*d* (Pre-12 m)
	M	SD	M	SD	M	SD	M	SD	Time	*p* value	Group	*p* value	GxT	*p* value			
A. SDQ self-report version																	
Emotional symptoms																	
UP-A	5.67	1.85	4.66	2.33	4.43	2.51	3.94	2.41	135.97	<0.0001	0.19	0.66	0.83	0.84	0.48	0.56	0.81
Control	5.75	1.94	4.73	2.37	4.32	2.58	4.19	2.73	–	–	–	–	–	–	0.47	0.63	0.66
Conduct problems																	
UP-A	2.66	1.69	2.40	1.80	2.02	1.59	1.58	1.65	44.66	<0.0001	3.08	0.08	1.26	0.74	0.15	0.39	0.65
Control	2.84	1.77	2.81	1.91	2.32	1.68	2.12	1.88	–	–	–	–	–	–	0.02	0.3	0.4
Peer relationship problems																	
UP-A	2.80	1.80	2.49	1.69	2.47	1.78	2.23	1.63	17.78	<0.0001	0.22	0.64	8.92	0.64	0.18	0.18	0.33
Control	2.67	1.65	2.81	1.94	2.46	1.66	2.63	1.75	–	–	–	–	–	–	0.08	0.13	0.02
Hyperactivity/inattention																	
UP-A	5.37	2.05	5.02	2.14	4.70	2.17	4.18	2.11	69.64	<0.0001	5.04	0.03	4.03	0.26	0.17	0.32	0.57
Control	5.74	1.88	5.33	2.09	4.85	2.12	5.03	2.27	–	–	–	–	–	–	0.21	0.44	0.34
Prosocial behavior																	
UP-A	8.20	1.61	8.17	1.78	8.35	1.60	8.50	1.78	32.02	<0.0001	0.06	0.82	0.90	0.83	0.02	0.09	0.17
Control	8.25	1.56	7.99	1.80	8.31	1.58	8.47	1.82	–	–	–	–	–	–	0.15	0.05	0.13
Total score																	
UP-A	16.50	4.21	14.58	5.49	13.61	5.69	11.93	5.02	163.24	<0.0001	3.91	0.048	3.67	0.30	0.39	0.58	0.99
Control	17.00	3.98	15.68	5.28	13.95	5.35	13.97	6.34							0.28	0.64	0.58
B. Parent version																	
Emotional symptoms																	
UP-A	4.26	1.97	3.97	1.63	3.75	1.73	3.61	1.64	32.33	<0.0001	1.47	0.23	0.64	0.89	0.16	0.13	0.08	
Control	4.64	1.96	4.26	1.67	3.92	1.64	3.71	1.72	–	–	–	–	–	–	0.21	0.20	0.12	
Conduct problems																	
UP-A	2.45	1.45	2.19	1.30	2.21	1.29	1.92	1.01	8.93	0.03	5.69	0.02	3.60	0.31	0.19	0.01	0.25	
Control	2.59	1.48	2.40	1.22	2.43	1.31	2.51	1.55	–	–	–	–	–	–	0.14	0.01	0.05	
Peer relationship problems																	
UP-A	2.81	1.81	2.49	1.53	2.47	1.40	2.23	1.26	16.86	0.001	0.43	0.51	2.20	0.53	0.18	0.18	0.33	
Control	2.67	1.66	2.81	1.56	2.46	1.61	2.63	1.38	–	–	–	–	–	–	0.08	0.13	0.02	
Hyperactivity/inattention																	
UP-A	4.02	2.17	3.78	1.89	3.48	1.95	3.37	1.49	17.31	0.001	2.83	0.09	1.19	0.76	0.11	0.16	0.06
Control	4.21	2.12	4.07	1.74	3.93	1.75	3.72	1.44	–	–	–	–	–	–	0.07	0.08	0.13
Prosocial behavior																	
UP-A	8.22	1.46	8.34	1.47	8.52	1.28	8.57	1.01	15.77	0.001	0.01	0.96	0.94	0.82	0.08	0.12	0.04
Control	8.25	1.56	7.99	1.80	8.31	1.58	8.47	1.82	–	–	–	–	–	–	0.17	0.02	0.18
Total score																	
UP-A	12.37	5.46	11.14	4.38	10.27	4.47	9.79	3.78	50.60	<0.0001	7.68	0.006	1.37	0.71	0.25	0.20	0.11
Control	13.44	5.17	12.39	4.48	11.68	4.43	11.45	4.32							0.22	0.16	0.05

*Note*: M, mean; SD, standard deviation; G, group; T, time.

In the SDQ Parents’ Version, GEE analyses revealed a significant effect of Time on scores in the five dimensions and in the Total Difficulties score (*p* < 0.0001). Cohen’s *d* effect sizes were small to medium for the UP-A group and small for the active control group (see Table 4). Pairwise comparisons showed significant improvements in the Total Difficulties score from pretreatment to posttreatment and at the 6- and 12-month follow-ups in both groups. Similarly, in both groups, statistically significant differences were observed between pretreatment and the 6- and 12-month follow-ups in Emotional symptoms, and between pretreatment and 12 months in Hyperactivity and Prosocial behavior. In the case of the UP-A group, significant differences were also identified between pretreatment and the 6-month follow-up in Peer relationship problems, Hyperactivity, and Prosocial behavior, and between pretreatment and the 12-month follow-up in Peer relationship problems and Conduct problems. At posttreatment, and at the 6- and 12-month follow-ups, the UP-A group showed significantly lower scores on the Total Difficulties score compared to the active control group. Furthermore, at the 12-month follow-up, the UP-A group obtained significantly lower scores than the active control group on the Conduct Problems scale.

The GEE analyses indicated a significant effect of Time on suicidal behavior (*p* < 0.0001), but no effect of the Treatment Group (*p* = 0.73) or interaction effect (*p* = 0.95) was found. Cohen’s *d* effect sizes comparing pretreatment with posttreatment and follow-ups were moderate for both groups (see Supplementary Table S6 and Supplementary Figure S2). Pairwise comparisons showed significant decreases in SENTIA scores from pretreatment to posttreatment and at the 6- and 12-month follow-ups in both groups.

### Secondary outcomes: Effects on psychological strengths

The GEE analyses revealed a statistically significant effect on both Positive Affect (*p* < 0.0001) and Negative Affect (*p* < 0.0001), with no statistically significant treatment group or interaction effect found (see Table 5). Effect sizes were moderate to large for both treatment groups. Pairwise comparisons revealed that both dimensions of the PANAS-C improved significantly from pretreatment to posttreatment and at the 6- and 12-month follow-ups in both groups, with no significant differences in either dimension or group between posttreatment and the 6-month follow-up.

**Table 5.** Descriptive statistics, GEE results for the effect of the intervention (UP-A versus active control) on PANAS-C, KS-10, self-esteem, and academic performance, effect sizes across the four time points Table 5. long description.

	Pretest		Posttest		Posttest 6 m		Posttest 12 m		Wald’s Chi-square						*d* (Pre-Post)	*d* (Pre-6 m)	*d* (Pre-12 m)
	M	SD	M	SD	M	SD	M	SD	Time	*p* value	Group	*p* value	GxT	*p* value			
Positive affect																	
UP-A	15.05	3.83	17.30	4.26	16.87	4.38	18.59	3.73	184.26	<0.0001	0.81	0.37	1.19	0.37	0.56	0.44	0.94
Control	15.36	4.12	17.24	4.19	17.38	4.04	18.22	4.50	–	–	–	–	–	–	0.45	0.49	0.66
Negative affect																	
UP-A	13.34	3.53	11.67	3.85	11.46	3.96	10.64	3.70	135.31	<0.0001	1.93	0.17	1.02	0.80	0.45	0.50	0.75
Control	13.74	3.75	12.29	4.48	11.51	4.35	11.51	4.56	–	–	–	–	–	–	0.35	0.55	0.54
KS-10																	
UP-A	30.77	5.90	33.93	6.92	33.59	7.40	43.56	4.92	1124.51	<0.0001	0.81	0.37	2.61	0.46	0.52	0.44	2.02
Control	31.26	6.55	33.91	6.72	34.72	7.22	42.93	6.05	–	–	–	–	–	–	0.41	0.52	1.7
Self-esteem																	
UP-A	24.35	4.77	26.82	5.46	28.43	5.54	29.90	5.46	289.22	<0.0001	0.71	0.40	3.88	0.94	0.48	0.79	1.09
Control	24.08	4.75	26.81	5.29	27.77	5.48	28.34	6.61	–	–	–	–	–	–	0.54	0.72	0.74
Academic performance																	
UP-A	6.80	1.64	6.64	1.63	6.64	1.56	6.89	1.77	5.22	0.16	1.26	0.26	12.94	0.005	0.09	0.09	0.05
Control	6.72	1.84	6.80	1.89	6.78	1.71	6.62	1.66	–	–	–	–	–	–	0.05	0.03	0.06

*Note*: M, mean; SD, standard deviation; G, group; T, time.

The GEE analyses revealed a statistically significant effect on both health-related quality of life (*p* < 0.0001) and self-esteem (*p* < 0.0001), with no significant Treatment Group or interaction effect found (see Table 5). Pairwise comparisons showed significant improvements in quality of life and self-esteem from pretreatment to posttreatment, as well as at the 6- and 12-month follow-ups in both groups. It is worth mentioning that the improvements remained significant between the 6- and 12-month follow-ups in both subjective quality of life and self-esteem in both groups. Although the Time factor was not statistically significant for academic performance, pairwise comparisons revealed statistically significant differences in the mean score between pretreatment and posttreatment (*p* = 0.0001), as well as at 6-month follow-up in the UP-A group (*p* = 0.0001). The effect sizes (Cohen’s *d*) were small for both groups (Table 5).

### Acceptability: Satisfaction with the interventions

As can be seen in Supplementary Table S7, participants in both treatment groups considered the workshop useful, applicable, and appropriate, reporting improvements in their perception of their ability to manage their emotions. Although the mean scores of the UP-A group were higher across the various indicators, these differences were not statistically significant, except for the recommendation of the workshop and the perception of the objectives set as realistic.

## Discussion

The aim of this study was to examine the effectiveness of the UP-A protocol in adolescents with subthreshold anxiety-depressive symptoms, compared to an active control group (protocolized progressive relaxation), including 6- and 12-month follow-up assessments. To our knowledge, the PSICE study is one of the largest worldwide RCTs conducted to test UP-A in adolescents at high risk for emotional disorder in non-clinical settings and delivered in a group format.

Regarding the primary indicators of self-reported depression and anxiety symptoms, both conditions showed improvement over time, with moderate to large effect sizes; however, no statistically significant differences were observed between conditions. The effects in the UP-A condition remained stable over time, with a pattern of sustained improvement and slightly higher effect sizes. This trend was most evident for depressive symptoms at the 12-month follow-up, which showed a marginally significant p-value (*p* = 0.056), suggesting a potential clinical benefit of the UP-A in maintaining therapeutic gains. At the 12-month follow-up, 78.5% of participants achieved ≥30% reduction in PHQ-9 scores; for GAD-7, 69.2% achieved ≥30% reduction. Overall, these findings align with previous evidence demonstrating the efficacy of the UP in addressing depressive and anxiety disorders and symptoms in adults, adolescents, and children (Carlucci et al., 2021; Cuijpers et al., 2023; Farchione et al., 2024; Newby et al., 2015; Sakiris & Berle, 2019; Schaeuffele et al., 2024). These results also converge with findings from Vivas-Fernandez et al. (2025), whose RCT comparing UP-A and an active control condition (e.g. emotional psychoeducation) likewise found no significant differences between the conditions on primary emotional health outcomes. These results are also consistent with findings from systematic reviews of universal and indicated school-based interventions (Gee et al., 2020; Rasing et al,. 2017; Wang et al., 2024). For instance, Gee et al. (2020) reported that indicated school-based interventions produce small effects for depression and moderate effects for anxiety in adolescents, with limited evidence of sustained benefits. Additionally, evidence suggests that relaxation-based strategies can also reduce emotional symptoms, supporting the value of complementary low-intensity approaches for promoting emotional health in youth (Hamdani et al., 2022).

The lack of differential efficacy between conditions may be partly explained by the activation of shared contextual and relational processes in both interventions. Factors such as regular adult attention, group structure, opportunities for emotional expression, peer normalization, and a safe and predictable setting may have contributed to improvement regardless of the specific therapeutic content. In addition, neither intervention directly targeted family dynamics or school-system variables, which are known to play a key role in adolescent emotional adjustment, potentially constraining the emergence of differential effects.

Regarding the secondary indicators, no statistically significant differences were observed between conditions; however, the effect sizes were generally larger in the UP-A condition, both in the transdiagnostic dimensions of emotional disorders and in positive and negative affect. The main differential effect observed was in the neuroticism dimension, where a marginally statistically significant difference was found in favor of the UP-A condition at the 12-month follow-up (*p* = 0.052). According to previous studies, the UP addresses shared mechanisms associated with neuroticism, in particular, negative evaluation and avoidance of intense emotional experience. A RCT has demonstrated that the UP significantly reduces levels of neuroticism in the adult population (Sauer-Zavala et al., 2020). In addition, neuroticism is a core personality domain with major public health implications, influencing a broad range of psychopathological disorders and physical health concerns. These results reinforce the relevance of neuroticism in the prevention of mental health and physical health problems (Carlucci et al., 2021; Farchione et al., 2024).

A key finding of this study is that the UP-A showed greater efficacy than the progressive relaxation condition on some externalizing outcomes. Significant improvements were observed in hyperactivity, conduct problems, and total mental health difficulties, which were maintained at the 12-month follow-up. These findings represent a relevant contribution, as most research on UP-A has focused on internalizing symptoms, leaving its impact on behavioral dimensions in school contexts less explored. This result is also consistent with the systematic review by Wang et al. (2024), which concludes that transdiagnostic interventions can generate broad benefits, including improvements in externalizing and behavioral adjustment variables. These results suggest that addressing core emotional processes such as experiential avoidance or emotional awareness may have an indirect effect on observable behavior, which is relevant for improving school and social adjustment. Although these differences are potentially informative, they should be interpreted with caution, given the absence of group differences in primary outcomes, the number of secondary comparisons conducted, and the exploratory nature of these analyses. Accordingly, these findings should be considered hypothesis-generating and warrant replication in future studies specifically powered to examine externalizing outcomes.

Both conditions reduced suicidal behaviors, with moderate effect sizes. It should be noted that most previous studies do not collect information on suicidal behavior, as this domain is often treated as an exclusion criterion. However, emerging evidence suggests that prevention programs originally developed to enhance socio-emotional competencies can produce downstream ‘crossover effects’, including unanticipated benefits on suicidal behavior among young people (Ayer et al., 2023). The UP-A targets core processes such as emotional awareness, cognitive flexibility, and adaptive emotion regulation, mechanisms that function as a central transdiagnostic factor in internalizing problems and key protective factors against self-injurious and suicidal behavior in adolescents (Al-Halabí & Fonseca-Pedrero, 2024). Interventions that strengthen emotion regulation strategies tend to reduce emotional reactivity, enhance adaptive coping, and improve behavioral control, which may partially explain the clinically meaningful reductions in suicidal behaviors observed in adolescents at high risk in our study (Kraft et al., 2023). Thus, the skills cultivated in the UP-A could confer broader ecological and clinical benefits beyond symptom reduction, contributing to safer behavioral profiles among vulnerable youth.

Both the UP-A and the progressive relaxation conditions showed improvements over time in terms of subjective quality of life and self-esteem. The effect sizes comparing pretreatment with the 12-month follow-up for quality of life and self-esteem were large, particularly in the UP-A group. However, such differences seem to emerge primarily during the follow-up period, as some variables did not show a significant pre-postintervention difference. To date, no studies using the UP-A have analyzed its impact on self-esteem. The PROCARE study found improvements in quality of life after the application of the UP-A in Spanish adolescents, with the impact remaining at 12 months with moderate effect sizes (Vivas-Fernandez et al., 2025).

These results have clear conceptual and practical implications. Transdiagnostic approaches aim to address the assumed shared mechanisms underlying psychological problems, such as neuroticism and associated emotional dysregulation. These underlying factors in mental health problems during childhood and adolescence are often transdiagnostic in nature. In this regard, the review conducted by Lynch et al. (2021) identified several key transdiagnostic risk factors for psychopathology in young people. The UP-A does not focus on single disorders approach but rather uses general cognitive-behavioral principles across the full range of emotional disorders. The core modules of the UP-A are mainly directed at negative affect and subsequent emotion-driven emotional coping (emotional avoidance). Therefore, the observed improvements in anxiety symptoms, depressive symptoms, or even behavioral problems may be associated with changes in neuroticism and maladaptive emotion regulation strategies (e.g. emotional avoidance). These results align with previous works suggesting that changes in experiential avoidance and distress tolerance tend to occur simultaneously with reductions in emotional symptoms. Overall, these results are significant, as they suggest that both structured interventions may act on relevant etiological factors, not only on comorbid and specific symptoms. However, the absence of clear differences between conditions indicates that these effects may not be uniquely attributable to protocol-specific mechanisms in this school-based context.

It is important to acknowledge that growing evidence indicates that adolescent emotional and behavioral change is shaped not only by individual skills but also by broader contextual influences (e.g. Jiménez et al., 2024). Adolescent emotional distress is not solely an individual phenomenon but emerges within a broader ecological system. Emotional symptoms are shaped by family relationships, peer dynamics, classroom environments, and school-level structures, all of which interact with individual vulnerabilities and influence both the onset and maintenance of difficulties. Recognizing these contextual influences is essential, as interventions focused exclusively on student-level skill acquisition may not fully address the systemic determinants of emotional well-being (Al-Halabí & Fonseca-Pedrero, 2024; Ciulin et al., 2026). Moreover, research increasingly shows that psychological improvement often results from common therapeutic factors – such as structured adult attention, emotional validation, the experience of being heard, and supportive group interactions – rather than from protocol-specific techniques alone. These nonspecific mechanisms have been identified as major contributors to therapeutic change across modalities (Cuijpers et al., 2019; Norcross & Lambert, 2019; Wampold & Imel, 2015), suggesting that both the UP-A and the relaxation condition may have activated similar relational and contextual processes, helping to explain the comparable improvements observed across groups. In this regard, the identification and expression of emotional difficulties may also be shaped by school practices and institutional contexts (Fazel et al., 2014; Weisz et al., 2017), which were not directly examined in the present study.

There are also several limitations of this study that warrant consideration. Among them, the high dropout rate stands out, suggesting the presence of possible experimental mortality that may affect the external validity and longitudinal interpretation of the results. Although attrition analyses indicated that dropout was primarily associated with age and not with baseline levels of depressive and anxiety symptoms, some degree of selective attrition cannot be ruled out. Sensitivity analyses using multiple imputation were consistent with the primary results, but the relatively high attrition rate may still introduce some uncertainty in the findings. Future research should explore the factors associated with this dropout to optimize retention strategies. In addition, the absence of blind assessment and reliance on self-reported measures may have introduced biases. Although youth mental health was assessed using the parent-reported SDQ, future studies would benefit from incorporating objective or behavioral data sources (e.g. observational indicators or digital behavioral measures) and evaluating contextual factors such as school climate and family support, which are known to influence emotional development and intervention outcomes. A further limitation is that the study may be underpowered, as the power calculation assumed a large effect size (*d* = 0.80), whereas school-based psychosocial interventions typically show small to moderate effects (Gee et al., 2020). An additional methodological consideration relates to data clustering. Interventions were delivered within schools, in small groups, and by different professionals, which introduces potential nesting effects (e.g. school-, group-, or therapist-level variance). Although GEE models were used to account for correlated observations, residual clustering effects cannot be fully ruled out and may have influenced the precision of the estimates. Furthermore, although the use of an active control group improves internal validity, the greater psychoeducational and experiential burden of content between conditions may have contributed, at least in part, to the observed advantages of the UP-A. Future research should examine the implementation of the program in vulnerable populations, its digital adaptation (iUP-A), and include longer-term follow-up.

## Conclusion

Emotional problems among young people constitute a major educational and public health challenge worldwide, given their high prevalence, substantial associated morbidity and mortality, and the profound long-term consequences they impose on individuals, families, and society at large. Taken together, the absence of significant group or interaction effects for the primary outcomes indicates that the benefits observed cannot be attributed to protocol-specific mechanisms alone. Rather, the findings support the effectiveness of structured, school-based group interventions more generally in adolescents with subthreshold emotional symptoms. These findings suggest that indicated preventive interventions, when implemented in non-clinical settings, may offer meaningful benefits for youth mental health. The promotion of well-being and the prevention of psychosocial difficulties constitute essential public health priorities.

## Supporting information

10.1017/S0033291726105443.sm001 Fonseca-Pedrero et al. supplementary material Fonseca-Pedrero et al. supplementary material

## Supplementary material

The supplementary material for this article can be found at http://doi.org/10.1017/S0033291726105443.

## Long descriptions

### Figure 1. Long description

A flowchart organized into four vertical stages: Enrollment, Allocation, Follow-up, and Analysis.

1. Enrollment: At the top, 715 individuals were evaluated for eligibility. An arrow points right to an Excluded box containing 39 individuals: 10 did not meet inclusion criteria, 20 declined to participate, and 9 for other reasons. The remaining 676 were randomized.

2. Allocation: The path splits into two groups.

- Left branch: 378 assigned to the U P dash A intervention; all 378 received the intervention.

- Right branch: 298 assigned to active control; 289 received the intervention.

3. Follow-up: This stage tracks experimental mortality at three intervals.

- U P dash A group: posttest n equals 163, 6 months n equals 181, 12 months n equals 271.

- Active control group: posttest n equals 92, 6 months n equals 106, 12 months n equals 182.

4. Analysis: The final stage at the bottom.

- U P dash A group: experimental mortality at posttest n equals 163, 6 months n equals 181, 12 months n equals 271.

- Active control group: 116 analyzed at 12 months and 0 excluded from analysis.

Navigate back to Figure 1..

### Table 1. Long description

The table is organized into four columns: Variable, U P dash A (n = 378), Active control (n = 298), and Overall (n = 676). It is divided into two main sections: Sociodemographic variables and Clinical variables.

Sociodemographic variables:

* Age (M [S D]): U P dash A is 13.78 (1.41), Active control is 13.75 (1.36), Overall is 13.77 (1.39).

* Sex (n [% women]): U P dash A is 277 (73%), Active control is 213 (71%), Overall is 490 (72%).

* Nationality (n [% Spanish]): U P dash A is 354 (94%), Active control is 268 (90%), Overall is 622 (92%).

* Socioeconomic status (M [S D]): U P dash A is 6.12 (1.90), Active control is 6.15 (1.95), Overall is 6.14 (1.92).

Clinical variables:

* Mental health history (n [% ever had a mental health problem]): U P dash A is 174 (46%), Active control is 127 (43%), Overall is 301 (45%).

* Psychologist history (n [% ever consulted a psychologist]): U P dash A is 130 (34%), Active control is 110 (37%), Overall is 240 (36%).

Note: M stands for mean and S D stands for standard deviation.

Navigate back to Table 1..

### Table 2. Long description

The table is organized into two main sections for P H Q 9 (depressive symptoms) and G A D 7 (anxiety symptoms).

Header Structure:

Columns include Pretest, Posttest, Posttest 6 m, and Posttest 12 m, each subdivided into M (Mean) and S D (Standard Deviation). These are followed by Wald’s Chi-square results for Time, Group, and G x T (Group by Time interaction) with associated p values. The final columns list effect sizes (d) for Pre-Post, Pre-6 m, and Pre-12 m.

Data for P H Q 9:

* U P A Group: Means decrease from 12.43 at Pretest to 7.11 at 12 m. Wald’s Chi-square for Time is 198.58 (p < 0.0001). Effect size d at 12 m is 1.29.

* Control Group: Means decrease from 12.47 at Pretest to 8.85 at 12 m. Effect size d at 12 m is 0.70.

Data for G A D 7:

* U P A Group: Means decrease from 12.48 at Pretest to 6.94 at 12 m. Wald’s Chi-square for Time is 255.20 (p < 0.0001). Effect size d at 12 m is 1.39.

* Control Group: Means decrease from 12.57 at Pretest to 8.41 at 12 m. Effect size d at 12 m is 0.90.

Note: M stands for mean, S D for standard deviation, G for group, and T for time.

Navigate back to Table 2..

### Figure 2. Long description

The image consists of two vertically stacked line graphs. Both graphs share a Y axis ranging from 0 to 16 in increments of 2 and an X axis with four time points: Pre, Post, 6 m, and 12 m.

Top Panel: Depressive symptoms.

Both groups start at a mean score of approximately 12.5 at Pre.

The U P A group (solid line with square markers) shows a steady linear decrease to approximately 9.5 at Post, 9 at 6 m, and 7 at 12 m.

The Active control group (dashed line with circle markers) follows a similar decrease to 9.5 at Post and 8.5 at 6 m, but then shows a slight increase to approximately 9 at 12 m.

Bottom Panel: Anxiety symptoms.

Both groups start at a mean score of approximately 12.5 at Pre.

The U P A group (solid line with square markers) decreases to approximately 9.5 at Post, 9 at 6 m, and 7 at 12 m.

The Active control group (dashed line with circle markers) decreases to 9.5 at Post and 8.5 at 6 m, followed by a slight increase to approximately 9 at 12 m.

Both graphs include vertical error bars at each data point.

Navigate back to Figure 2..

### Table 3. Long description

The table presents descriptive statistics and G E E results for nine dimensions: Neuroticism, Positive temperament, Depressed mood, Autonomic arousal, Somatic anxiety, Social anxiety, Intrusive cognitions, Traumatic re-experiencing, and Avoidance.

For each dimension, data is split between U P dash A and Control groups across four time points: Pretest, Posttest, Posttest 6 m, and Posttest 12 m.

Key statistical columns include:

* Wald’s Chi-square for Time, Group, and Group by Time (G x T) interaction.

* p values for each Wald’s test.

* Cohen’s d effect sizes for Pre-Post, Pre-6 m, and Pre-12 m.

Notable trends:

* Neuroticism: U P dash A mean decreased from 21.48 at Pretest to 15.66 at 12 m. Wald’s Time p value is less than 0.0001.

* Depressed mood: U P dash A mean decreased from 15.39 at Pretest to 9.13 at 12 m. Wald’s Time p value is less than 0.0001.

* Intrusive cognitions: U P dash A mean decreased from 19.06 at Pretest to 11.03 at 12 m. Wald’s Time p value is less than 0.0001.

* Positive temperament: Both groups showed increases over time, with U P dash A rising from 22.53 to 25.89.

* Somatic anxiety: Showed a significant time effect (p = 0.004) but smaller effect sizes compared to other dimensions.

Across all dimensions, the Wald’s Chi-square for Time was significant (p < 0.05), while Group and G x T interactions generally did not reach statistical significance.

Navigate back to Table 3..

### Table 4. Long description

The table is divided into two main sections: A. S D Q self-report version and B. Parent version. Each section tracks five dimensions (Emotional symptoms, Conduct problems, Peer relationship problems, Hyperactivity/inattention, Prosocial behavior) and a Total score.

For each dimension, the table provides:

* Means (M) and Standard Deviations (S D) for Pretest, Posttest, 6-month Posttest, and 12-month Posttest.

* Wald’s Chi-square results for Time, Group, and Group-by-Time (G x T) interactions with associated p-values.

* Effect sizes (d) for Pre-Post, Pre-6 month, and Pre-12 month intervals.

Key data points from Section A (Self-report):

* Emotional symptoms: U P-A mean decreased from 5.67 at pretest to 3.94 at 12 months. Time effect p-value is less than 0.0001.

* Total score: U P-A mean decreased from 16.50 at pretest to 11.93 at 12 months. Control mean decreased from 17.00 to 13.97. Group effect p-value is 0.048.

Key data points from Section B (Parent version):

* Conduct problems: U P-A mean decreased from 2.45 to 1.92. Group effect p-value is 0.02.

* Total score: U P-A mean decreased from 12.37 at pretest to 9.79 at 12 months. Control mean decreased from 13.44 to 11.45. Group effect p-value is 0.006.

Across most dimensions in both versions, the Time effect is highly significant (p < 0.001), indicating improvement over time for both groups, with U P-A often showing larger effect sizes (d) by the 12-month mark compared to the control group.

Navigate back to Table 4..

### Table 5. Long description

The table presents descriptive statistics and G E E results for five outcome measures: Positive affect, Negative affect, K S dash 10, Self-esteem, and Educational performance. Data is provided for U P dash A and Control groups across four time points: Pretest, Posttest, Posttest 6 m, and Posttest 12 m.

* Positive affect: U P dash A mean increased from 15.05 to 18.59. Wald’s Chi-square for Time was 184.26 p < 0.0001.

* Negative affect: U P dash A mean decreased from 13.34 to 10.64. Wald’s Chi-square for Time was 135.31 p < 0.0001.

* K S dash 10: Both groups showed significant increases over time, with U P dash A reaching 43.56 at 12 m. Wald’s Chi-square for Time was 1124.51 p < 0.0001.

* Self-esteem: U P dash A mean increased from 24.35 to 29.90. Wald’s Chi-square for Time was 289.22 p < 0.0001.

* Educational performance: U P dash A mean remained stable around 6.8. This was the only measure with a significant Group by Time interaction G x T Wald’s Chi-square of 12.94 p = 0.005.

Effect sizes d are provided for Pre-Post, Pre-6 m, and Pre-12 m comparisons for all measures.

Navigate back to Table 5..
