# Supplementary material for: A multicenter randomized controlled effectiveness trial of transdiagnostic treatment for adolescents with subthreshold anxiety and depressive symptoms
Source: Psychol Med. 2026 Sep 4;56:e264. doi: 10.1017/S0033291726105443 (PMC13735848; doi:10.1017/S0033291726105443)
Supplement: Supplementary file 1 [file S0033291726105443sup001.docx]

**Supplementary Materials**

**Appendix A: Methods Supplement**

Supplementary Table S1: Overview of the UP-A Adapted for Preventive Intervention	on PSICE Project	Page 2

Supplementary Table S2: Content and Sessions in the Active Control Group: Progressive	Relaxation Training	Page 3

Supplementary Table S3: Dropout Rate for the UP-A and Active Control Groups Throughout the Different Assessment Stages	Page 4

Supplementary Table S4: CONSORT Checklist	Page 5

**Appendix B: Results Supplement**

Supplementary Table S4: CONSORT Checklist	Page 5

Supplementary Table S5: Baseline Characteristics of Completers and Non-completers at 12-month follow-up for the UP-A and the Active Control Groups

Supplementary Table S6: Descriptive Statistics, GEE Results for the Effect of the	Intervention (UP-A vs. Active Control) on Suicidal Behavior	Page 8

Supplementary Table S7: Descriptive Statistics for Satisfaction with Both Conditions: UP-A vs. Active Control	Page 9

Figure S1: Evolution of the Means in the Evaluated Dimensions of the MEDI	Page 10

Figure S2: Mean Scores on Suicidal Behavior Measured by SENTIA	Page 14

**Appendix A: Methods Supplement**

**Table S1**

*Overview of the UP-A Adapted for Preventive Intervention on PSICE Project (Adapted and Modified from).*

| Number Sessions | Module title | Main content |
| --- | --- | --- |
| 1 | 1.Building and Keeping Motivation | Introduce confidentiality and group rules; obtain 3 top problems, severity ratings, and a SMART (specific, measurable, attainable, relevant, and time-bound) goal for each problem; complete emotion identification skills activity if sufficient time remains. |
| 1 | 2.Getting to Know Your Emotions and Behaviors | Psychoeducation about emotions and their function; introduce emotional behaviors, the 3 parts of an emotional experience, and the “Before, During, and After” form for tracking emotional experiences outside of sessions. |
| 1 | 3.Emotion-Focused Behavioral Experiments | Psychoeducation about cycle of avoidance, opposite action, and behavioral experiments; reflect on current use of free time and come up with a list of enjoyed activities; introduce weekly activity tracker for on-going behavioral activation. |
| 1 | 4.Awareness of Physical Sensations | Psychoeducation about body sensations, their relationship to intense emotions and their harmlessness; introduce the concept of “fight or flight response” and review cycle of avoidance; conduct sensational exposures with the group. |
| 2 | 5.Being Flexible in Your Thinking | Introduce the concept of “thinking traps” (i.e., cognitive distortions) and teach common thinking traps; introduce the concept of automatic and alternative thoughts as well as detective thinking skills; Re-rate top problems obtained in session 1.  Review thinking traps and detective thinking skills; introduce and ensure understanding of problem-solving skills; conduct examples using problem solving skills with group members; review skills learnt so far in the program. |
| 1 | 6.Awareness of Emotional Experiences | Introduce the rationale for present-moment awareness and practice this skill in session using non-emotional stimuli (e.g., focus on breathing); introduce rationale for non-judgmental awareness; do an individual mini-test assessing skills taught in the program so far. |
| 2 | 7.Situational Emotion Exposures | Review cycle of avoidance, reinforcement, and maintenance of learned behavior; provide psychoeducation about emotion exposures; create emotional behaviors forms to identify relevant exposures; if time permits, conduct a group exposure activity; assign exposure homework. |
| 1 | 8. Keeping it Going Maintaining Your Gains | Review exposure homework and plan future exposures if necessary; re-rate top problems and revisit SMART goals; review skills that have been most useful for each group member and make an individualized post-program plan to practice skills. |
| 2 | Module-P: Parenting the Emotional Adolescent | Introduce parents to increase parents’ awareness of their own emotional responses to adolescent distress; introduce parents to four emotional parenting behaviors that are generally thought to be ineffective or less effective ways of responding to adolescent distress. |

**Table S2**

*Content and Sessions in the Active Control Group: Progressive Relaxation Training*

| Module Name | Module  Description | Session |
| --- | --- | --- |
| 1 | Basic procedure for 16 muscle groups (tension-relaxation) | 1, 2 and 3 |
| 2 | Basic procedure for 7 muscle groups (tension-relaxation) | 4 and 5 |
| 3 | Basic procedure for 4 muscle groups (tension-relaxation) | 6 and 7 |
| 4 | Basic procedure for 4 muscle groups (mental journey, without tension exercises) | 8 |
| 5 | Basic procedure for 4 muscle groups (mental journey and counting backwards as a technique for achieving rapid relaxation) | 9 |
| 6 | Basic procedure for 4 muscle groups (count backwards) | 10 |

**Table S3**

*Dropout Rate for the UP-A and Active Control Groups Throughout the Different Assessment Stages*

| Time | UP-A | | Active control | | Total | |
| --- | --- | --- | --- | --- | --- | --- |
|  | *n* | % Dropout rate | *n* | % Dropout rate | *N* | % Dropout rate |
| Pretreatment | 378 | -- | 298 | -- | 676 | -- |
| Posttreatment | 215 | 43.12 | 206 | 30.87 | 421 | 37.72 |
| 6-month follow-up | 197 | 47.88 | 192 | 35.57 | 389 | 42.46 |
| 12-month follow-up | 107 | 71.69 | 116 | 61.07 | 223 | 67.01 |

**Table S4**

*CONSORT Checklist*

| Section/topic | No | CONSORT 2025 checklist item description | Reported on page no. |
| --- | --- | --- | --- |
| **Title and abstract** | | |  |
| Title and structured abstract | 1a | Identification as a randomised trial | 1 |
|  | 1b | Structured summary of the trial design, methods, results, and conclusions | 2 |
| **Open science** | | |  |
| Trial registration | 2 | Name of trial registry, identifying number (with URL) and date of registration | 4 |
| Protocol and statistical analysis plan | 3 | Where the trial protocol and statistical analysis plan can be accessed | Reference 25 |
| Data sharing | 4 | Where and how the individual de-identified participant data (including data dictionary), statistical code and any other materials can be accessed | N/A |
| Funding and conflicts of interest | 5a | Sources of funding and other support (eg, supply of drugs), and role of funders in the design, conduct, analysis and reporting of the trial | 7 |
|  | 5b | Financial and other conflicts of interest of the manuscript authors | 14/15 |
| **Introduction** | | |  |
| Background and rationale | 6 | Scientific background and rationale | 3/4 |
| Objectives | 7 | Specific objectives related to benefits and harms | 4 |
| **Methods** | | |  |
| Patient and public involvement | 8 | Details of patient or public involvement in the design, conduct and reporting of the trial | 5/6 |
| Trial design | 9 | Description of trial design including type of trial (eg, parallel group, crossover), allocation ratio, and framework (eg, superiority, equivalence, non-inferiority, exploratory) | 4/5 |
| Changes to trial protocol | 10 | Important changes to the trial after it commenced including any outcomes or analyses that were not prespecified, with reason | N/A |
| Trial setting | 11 | Settings (eg, community, hospital) and locations (eg, countries, sites) where the trial was conducted | 4/5 |
| Eligibility criteria | 12a | Eligibility criteria for participants | 4/5 |
|  | 12b | If applicable, eligibility criteria for sites and for individuals delivering the interventions (eg, surgeons, physiotherapists) | 6 |
| Intervention and comparator | 13 | Intervention and comparator with sufficient details to allow replication. If relevant, where additional materials describing the intervention and comparator (eg, intervention manual) can be accessed | 5/6 |
| Outcomes | 14 | Prespecified primary and secondary outcomes, including the specific measurement variable (eg, systolic blood pressure), analysis metric (eg, change from baseline, final value, time to event), method of aggregation (eg, median, proportion), and time point for each outcome | 6/7 |
| Harms | 15 | How harms were defined and assessed (eg, systematically, non-systematically) | 6 |
| Sample size | 16a | How sample size was determined, including all assumptions supporting the sample size calculation | 6/7 |
|  | 16b | Explanation of any interim analyses and stopping guidelines | N/A |
| Randomisation: |  |  |  |
| Sequence generation | 17a | Who generated the random allocation sequence and the method used | 5 |
|  | 17b | Type of randomisation and details of any restriction (eg, stratification, blocking and block size) | 5 |
|  |  |  | **Reported on page no.** |
| Allocation concealment mechanism | 18 | Mechanism used to implement the random allocation sequence (eg, central computer/telephone; sequentially numbered, opaque, sealed containers), describing any steps to conceal the sequence until interventions were assigned | 5 |
| Implementation | 19 | Whether the personnel who enrolled and those who assigned participants to the interventions had access to the random allocation sequence | 5 |
| Blinding | 20a | Who was blinded after assignment to interventions (eg, participants, care providers, outcome assessors, data analysts) | 5 |
|  | 20b | If blinded, how blinding was achieved and description of the similarity of interventions | N/A |
| Statistical methods | 21a | Statistical methods used to compare groups for primary and secondary outcomes, including harms | 7/8 |
|  | 21b | Definition of who is included in each analysis (eg, all randomised participants), and in which group | 8/9 |
|  | 21c | How missing data were handled in the analysis | 8 |
|  | 21d | Methods for any additional analyses (eg, subgroup and sensitivity analyses), distinguishing prespecified from post hoc | 8 |
| **Results** | | |  |
| Participant flow, including flow diagram | 22a | For each group, the numbers of participants who were randomly assigned, received intended intervention, and were analysed for the primary outcome | Figure 1 |
|  | 22b | For each group, losses and exclusions after randomisation, together with reasons | Figure 1/ Table S3 |
| Recruitment | 23a | Dates defining the periods of recruitment and follow-up for outcomes of benefits and harms | 5 |
|  | 23b | If relevant, why the trial ended or was stopped | N/A |
| Intervention and comparator delivery | 24a | Intervention and comparator as they were actually administered (eg, where appropriate, who delivered the intervention/comparator, how participants adhered, whether they were delivered as intended (fidelity)) | 5/6 |
|  | 24b | Concomitant care received during the trial for each group | N/A |
| Baseline data | 25 | A table showing baseline demographic and clinical characteristics for each group. |  |
| Numbers analysed,  outcomes and estimation | 26 | For each primary and secondary outcome, by group:  ● the number of participants included in the analysis  ● the number of participants with available data at the outcome time point  ● result for each group, and the estimated effect size and its precision (such as 95% confidence interval)  ● for binary outcomes, presentation of both absolute and relative effect size | 8-11 |
| Harms | 27 | All harms or unintended events in each group | N/A |
| Ancillary analyses | 28 | Any other analyses performed, including subgroup and sensitivity analyses, distinguishing pre-specified from post hoc | 8-11 |
| **Discussion** | | |  |
| Interpretation | 29 | Interpretation consistent with results, balancing benefits and harms, and considering other relevant evidence | 11-14 |
| Limitations | 30 | Trial limitations, addressing sources of potential bias, imprecision, generalisability, and, if relevant, multiplicity of analyses | 14 |

**Appendix B: Results Supplement**

**Table S5**

|  | UP-A | | |  | Active control | | |
| --- | --- | --- | --- | --- | --- | --- | --- |
| Variable | Completers (n=107) | Non-completers (n=271) | *p* |  | Completers (n=116) | Non-completers (n=182) | *p* |
| Female, n (%) | 75 (70.1%) | 202 (74.5%) |  |  | 82 (70.7%) | 131 (72%) |  |
| Spanish, n (%) | 103 (96.3%) | 251 (92.6%) |  |  | 104 (89.7%) | 164 (90.1%) |  |
| Age, M (SD) | 13.42 (1.27) | 13.93 (1.44) | .001 |  | 13.52 (1.12) | 13.90 (1.48) | .013 |
| SES, M (SD) | 6.48 (1.80) | 6.29 (1.93) |  |  | 6.09 (2.07) | 6.19 (1.87) |  |
| PHQ-9, M (SD) | 12.13 (2.99) | 12.55 (3.21) |  |  | 12.75 (3.68) | 12.30 (3.25) |  |
| GAD-7, M (SD) | 12.33 (2.85) | 12.54 (3.08) |  |  | 12.59 (3.00) | 12.56 (2.97) |  |

*Baseline Characteristics of Completers and* ***N****on-completers at 12-month follow-up for the UP-A and the Active Control Groups*

*Note.* Completers = participants who provided data at 12-month follow-up. Continuous variables compared using independent-samples *t* tests; categorical variables using *χ²* tests. Only statistically significant *p* values (*p* < .05) are reported. SES = socioeconomic status; PHQ-9 = Patient Health Questionnaire-9; GAD-7 = Generalized Anxiety Disorder-7.

**Table S6**

*Descriptive Statistics, GEE Results for the Effect of the Intervention (UP-A vs. Active Control) on Suicidal Behavior, Measured by SENTIA, and Effect Sizes Across the Four Time Points*

|  |  | Pretest | | Postest | | Postest 6m | | Postest 12m | | Wald's Chi-square | | | | *d*  *(Pre-Post)* | *d*  *(Pre-6m)* | *d*  *(Pre-12m)* |
| --- | --- | --- | --- | --- | --- | --- | --- | --- | --- | --- | --- | --- | --- | --- | --- | --- |
|  |  | *M* | *SD* | *M* | *SD* | *M* | *SD* | *M* | *SD* | Time | p value | Group | GxT |  |  |  |
|  | UP-A | 1.03 | 1.34 | 0.63 | 1.16 | 0.56 | 1.21 | 0.47 | 1.04 | 54.88 | <0.0001 | 0.12 | 0.35 | 0.32 | 0.37 | 0.47 |
|  | Control | 1.06 | 1.46 | 0.73 | 1.35 | 0.68 | 1.33 | 0.64 | 1.35 | -- | -- | -- | -- | 0.24 | 0.28 | 0.30 |

*Note*. *M* = Mean; *SD* = Standard deviation; G = Group; T = time.

**Table S7**

*Descriptive Statistics for Satisfaction with Both Conditions: UP-A vs. Active Control*

| A. Range of scores 1-10 | | *M* | *SD* | *F* | *p value* |
| --- | --- | --- | --- | --- | --- |
| 1. How much have you learned from this workshop? | |  |  |  |  |
|  | UP-A | 7.50 | 1.73 | 0.28 |  |
|  | Control | 7.39 | 2.02 |  |  |
| 2. To what extent has it helped you cope with life in general? | |  |  |  |  |
|  | UP-A | 7.06 | 1.93 | 0.94 |  |
|  | Control | 6.85 | 2.17 |  |  |
| 3. How much did you enjoy participating in this workshop? | |  |  |  |  |
|  | UP-A | 7.84 | 2.06 | 3.21 |  |
|  | Control | 7.42 | 2.42 |  |  |
| 4. What was your ability to manage your emotions before the workshop? | |  |  |  |  |
|  | UP-A | 4.57 | 2.05 | 1.17 |  |
|  | Control | 4.82 | 2.41 |  |  |
| 5. What is your ability to manage your emotions after the workshop? | |  |  |  |  |
|  | UP-A | 7.27 | 1.70 | 0.35 |  |
|  | Control | 7.16 | 2.00 |  |  |
| 6. Change in ability to manage emotions before and after the workshop | |  |  |  |  |
|  | UP-A | 2.69 | 2.08 | 2.22 |  |
|  | Control | 2.35 | 2.27 |  |  |
| B. Yes/no | | *n* | Yes (%) | *χ^2^* | *p value* |
| 7. Has this workshop helped you learn more about emotions and how they work? | |  |  |  |  |
|  | UP-A | 180 | 97.3% | 2.08 |  |
|  | Control | 164 | 94.3% |  |  |
| 8. Would you recommend this workshop to other kids your age? | |  |  |  |  |
|  | UP-A | 181 | 97.8% | 9.42 | <0.01 |
|  | Control | 157 | 90.2% |  |  |
| C. Range of scores 1-5 | | *M* | *SD* | *F* | *p value* |
| 9. I liked this workshop | |  |  |  |  |
|  | UP-A | 4.26 | 0.86 | 2.36 |  |
|  | Control | 4.11 | 1.06 |  |  |
| 10. This workshop was appropriate | |  |  |  |  |
|  | UP-A | 4.34 | 0.82 | 2.37 |  |
|  | Control | 4.19 | 0.97 |  |  |
| 11. This workshop is easy to apply | |  |  |  |  |
|  | UP-A | 4.05 | 0.91 | 1.67 |  |
|  | Control | 3.92 | 1.06 |  |  |
| 12. This workshop sets realistic goals | |  |  |  |  |
|  | UP-A | 4.41 | 0.80 | 6.41 | <0.01 |
|  | Control | 4.14 | 1.04 |  |  |

*Note*. *M* = Mean; *SD* = Standard deviation; G = Group; T = time.

**Figure s1**

*Evolution of the Means in the Evaluated Dimensions of the MEDI Throughout the Four Times Evaluated in the Two Intervention Groups (UP-A vs. Active Control)*

Neuroticism

|  | UPA | Active control |
| --- | --- | --- |
| Pre | 21.4771 | 22.4915 |
| Post | 19.2186 | 19.2816 |
| 6m | 17.9695 | 19.3385 |
| 12m | 15.6636 | 18.181 |

Positive temperament

|  | UPA | Active control |
| --- | --- | --- |
| Pre | 22.5286 | 21.5769 |
| Post | 24.186 | 23.4709 |
| 6m | 24.4264 | 24.3229 |
| 12m | 25.8879 | 25.0862 |

Depressed mood

|  | UPA | Active control |
| --- | --- | --- |
| Pre | 15.3857 | 16.0299 |
| Post | 12.2 | 12.432 |
| 6m | 10.5381 | 11.4635 |
| 12m | 9.13208 | 10.8017 |

Autonomic arousal

|  | UPA | Active control |
| --- | --- | --- |
| Pre | 13.4286 | 13.0726 |
| Post | 10.1116 | 10.6019 |
| 6m | 9.32995 | 9.22396 |
| 12m | 8.46226 | 9.31897 |

Somatic anxiety

|  | UPA | Active control |
| --- | --- | --- |
| Pre | 14.6257 | 14.8205 |
| Post | 14.4372 | 14.8641 |
| 6m | 13.4873 | 14.7135 |
| 12m | 12.6509 | 14.1466 |

Social anxiety

|  | UPA | Active control |
| --- | --- | --- |
| Pre | 16.1257 | 16.5214 |
| Post | 14.2186 | 15.699 |
| 6m | 13.5381 | 14.0938 |
| 12m | 12.4151 | 14.4483 |

Intrusive cognitions

|  | UPA | Active control |
| --- | --- | --- |
| Pre | 19.0629 | 19.6709 |
| Post | 14.1674 | 15.6602 |
| 6m | 13.264 | 13.75 |
| 12m | 11.0283 | 13.2155 |

Traumatic re-experiencing

|  | UPA | Active control |
| --- | --- | --- |
| Pre | 14.2257 | 13.9785 |
| Post | 10.6512 | 11.1893 |
| 6m | 10.1421 | 9.58854 |
| 12m | 7.50943 | 8.57759 |

Avoidance

|  | UPA | Active control |
| --- | --- | --- |
| Pre | 25.1429 | 24.5021 |
| Post | 22.8047 | 23.7573 |
| 6m | 21.4975 | 21.6667 |
| 12m | 19.9057 | 20.7241 |

**Figure S2**

*Mean Scores on Suicidal Behavior, Measured by SENTIA test, Throughout the Four Assessment Periods in the Two Intervention Groups (UP-A vs. Active Control***)**

Suicidal behavior

|  | UPA | Active control |
| --- | --- | --- |
| Pre | 1.03439 | 1.06376 |
| Post | 0.632558 | 0.728155 |
| 6m | 0.563452 | 0.677083 |
| 12m | 0.46729 | 0.637931 |
